# THE INFLUENCE OF PASSIVE TOBACCO EXPOSURE AND PHYSICAL EXERCISE ON BONE TISSUE OF YOUNG RATS

**DOI:** 10.1590/1413-785220172502130777

**Published:** 2017

**Authors:** Regina Celi Trindade Camargo, Regiane Rocha Costalonga, Mário Jefferson Quirino Louzada, Rômulo Araújo Fernandes, José Carlos Silva Camargo, Jacqueline Bexiga Urban

**Affiliations:** 1 Universidade Estadual Paulista, Faculdade de Ciências e Tecnologia, Presidente Prudente, SP, Brazil.; 2 Universidade Estadual Paulista, Faculdade de Medicina Veterinária, Araçatuba, SP, Brazil.

**Keywords:** Exercise, Tobacco smoke pollution, Rats, Wistar/growth & development, Lactation, Birth weight

## Abstract

**Objective:**

The objective of this study is to investigate the effect of passive smoking during pregnancy and associated with swimming on bone area growth, bone mineral density (BMD), and bone mineral content (BMC).

**Methods:**

The offspring was grouped by control matrices (G1) and passive smokers (G2). The offspring was regrouped in eight subgroups, with exposure to smoking (2x/day) and physical exercise (1 session/day), respecting the group of matrices in: sedentary control (G1CS and G2CS), swimming control (G1CN and G2CN), sedentary passive smoker (G1FS and G2FS), and passive smoker swimmer (G1FN and G2FN). The area, BMD and BMC were measured by the tibia and femur and analyzed by densitometer. The results were analyzed by One-Way ANOVA test with Tukey post-test, with a significance level of 5%.

**Results:**

In the tibia BMC study, a better rate was observed in G2CN group when compared to G1CS, G1CN and G1FN (p ≤ 0.023). When assessing BMD in the femur, a higher density ratio was observed in G1FS group when compared to G2CS, G2CN, G2FS and G2FN (p<0.008). In the tibia study, the animals of the G1FS group had higher rates when compared to G2CS and G2FN groups (p≤0.007).

**Conclusions:**

The model of male offspring exposed to passive smoking during fetal development showed a strong decrease in the analyzed parameters. ***Level of Evidence I, Randomized High Quality Clinical Trial With or Without Statistically Significant Difference, But Narrow Confidence Intervals.***

## RESUMO

**Objetivo:**

O objetivo desse estudo é investigar o efeito do tabagismo passivo durante a prenhez e associado à natação no crescimento de área óssea, densidade mineral óssea (DMO) e conteúdo mineral ósseo (CMO).

**Métodos:**

Os filhotes foram gerados por matrizes controle (G1) e tabagistas (G2). Os filhotes foram reagrupados em oito subgrupos, com exposição ao tabagismo (2x/dia) e realização de exercício físico (1 sessão/dia), respeitando o grupo de matrizes em: controle sedentário (G1CS e G2CS), controle natação (G1CN e G2CN), tabagista passivo sedentário (G1FS e G2FS) e tabagista passivo natação (G1FN e G2FN). A área, a DMO e o CMO foram aferidos pela tíbia e pelo fêmur e analisados pelo densitômetro. Os resultados foram analisados pelo teste de ANOVA One-Way, com pós-teste de Tukey, com nível de significância de 5%.

**Resultados:**

No estudo do CMO da tíbia, foi observada taxa melhor no grupo G2CN quando comparada aos filhotes dos grupos G1CS, G1CN e G1FN (p ≤ 0,023). Na averiguação da DMO no fêmur, foi observada maior taxa de densidade no grupo G1FS quando confrontado aos grupos G2CS, G2CN, G2FS e G2FN (p ≤ 0,008). Já no estudo da tíbia, os animais do grupo G1FS apresentaram maiores taxas quando comparados aos grupos G2CS e G2FN (p≤0,007).

**Conclusões:**

O modelo de filhotes machos expostos ao tabagismo passivo durante o desenvolvimento fetal apresentou uma forte diminuição dos parâmetros analisados. Nível de Evidência I, Estudo Clínico Randomizado de Alta Qualidade Com ou Sem Diferença Estatisticamente Significante, Mas Com Intervalos de Confiança Estreitos.

## INTRODUCTION

Exercise is known to have an important role in bone development.^1^ There is also a known association between physical exercise and reduced bone resorption and bone formation, and exercise is consequently a strategy to reduce bone fragility and fracture risk, which are very common in childhood.¹ Notable among clinical indicators of bone health are bone mineral content (BMC) and bone mineral density (BMD).^2^ Weight-bearing physical activity is reported to encourage increases in BMC and BMD, but there is no consensus about non-weight-bearing exercise such as swimming.^2^

In contrast to the benefits of physical exercise, the effects of nicotine (a component of cigarettes) are indicated as a factor in bone tissue deterioration,^3^ and appear to be associated with the dose-response mechanism.^4^

Finally, human and rat skeletons are known to exhibit similar responses to mechanical and hormonal influences as well as the effects of other agents.^2^ This study consequently hypothesizes that passive smoking could negatively impact rat growth from gestation to adulthood, and that its influence could be minimized by physical exercise.

In this way we intend to evaluate bone formation during passive exposure to cigarette smoke during gestation and in association with physical exercise during growth by measuring BMC and BMD.

## METHODS

This study used the technical experimental procedure approved by the institutional review board of the Faculdade de Ciências e Tecnologia da Universidade Estadual Paulista "Julio de Mesquita Filho", UNESP, Campus de Presidente Prudente under process 06/2011, and followed the ethical principles for animal experimentation adopted by the Sociedade Brasileira de Ciência em Animais de Laboratório (SBCAL).

Initially 15 animals were used, 11 virgin female and 4 male (72 days old) Wistar rats (*Rattus novergicus*, var. Albino, Rodentia, Mammalia). They were kept in individual cages at an average temperature of 22 ± 2°C, humidity of 50 ± 10%, 12-hour light/dark cycle (7 am-7 pm), and had free access to water and standard rat chow.

The rats were divided into two groups: G1 (control group, n=5) and G2 (group exposed to cigarette smoke, n=6). The exposure protocol began on the 72nd day of life for the breeding females and ended when lactation ended.

At the 90th day of the life, a vaginal swab was performed on the female rats to verify the phase of the estrous cycle; if estrus was observed, the female rats were placed in individual cages with a male rat, where they stayed for one night for copulation. The next morning, pregnancy was diagnosed by the presence of sperm in the vaginal smear, which characterized day zero of the pregnancy.^5^

The male rat pups were kept with the mothers until the end of lactation, on their 21st day of life, and were subsequently divided into subgroups according to their parentage.

The rats were randomly divided into groups that were subjected to a maximum of two protocols (protocols for exposure to smoking and physical exercise): G1FN (n=14) and G2FN (n=15) (exercise group exposed to cigarette smoke); G1FS (n=10) and G2FS (n=09) (sedentary group exposed to cigarette smoke); G1CN (n=11) and G2CN (n=10) (exercise group, control); and G1CS (n=08) and G2CS (n=10) (sedentary group, control).

The protocol for adaptation to water started during the pups' fifth week of life, and in the sixth week they began the protocol for physical exercise and exposure to cigarette smoke.

There were two separate study periods during which the rats were exposed to cigarette smoke. The first period occurred when the female rats reached 72 days of life, and the second on the same day as the sixth swimming session for the rat pups.

The cigarette smoke exposure protocol was divided into two stages:

- Adaptation stage: the first five days of exposure to cigarette smoke for group G1 in the smoke chamber at a temperature of 23±1°C^6^ for 10 minutes, once a day, with 250 ppm (parts per million) of CO (carbon monoxide) measured using a specific gas sensor (ToxiPro device, Biosystems, Prairieville, United States); and

- Experimental stage: this stage began at the breeding females' 72nd day of life and ended at the end of lactation for the G2 group, and from the 6th session until the 21st day of lactation for the pups, with a session lasting 30 minutes, twice a day (morning and afternoon), five days per week, with 350 ppm CO per exposure.^7^

For better assimilation to the consumption of chronic smokers, the experimental dose totaled an average of two cigarettes/day/animal.^5^ The animals in the G1 group inhaled compressed air with the same time and frequency characteristics as the animals which were exposed to smoke.

For this protocol two hermetically sealed chambers were used: one for the control groups, which only inhaled compressed air, and a second box for the groups exposed to cigarette smoke. The smoke inhalation chamber was divided into two compartments: one in which lit cigarettes were placed, and another which held a cage with six animals, adapted from the inhalation model described by Cendon et al.^8^

Commercially acquired cigarettes were used, containing tobacco blends, sugars, cigarette paper, plant extracts, and flavoring agents, which for each lit cigarette produced: 9.3 ± 0.93 mg/cig tar,

0.78 ± 0.078 mg/cig nicotine, and 8.0 ± 1.2 mg/cig carbon monoxide, as stated on the product packaging.

The swimming program began during the fifth week of life for the male offspring. As described by Volpato et al.,^9^ we used a tank containing water at 30°C and a depth of 40 cm so that the pups could not reach the bottom of the tank with their tails, thus stimulating swimming.

The program was divided into two phases:

- adaptation to exercise: this comprised the five exercise sessions, with a progressive increase of 10 minutes' duration per day, starting with 20 minutes and lasting 60 minutes by the fifth session;
- exercise: from the sixth session, which lasted 60 minutes, to the 30th session, without additional extension of the training time.^10^

The exercise sessions took place every evening without interruption five days per week¹¹ for 30 sessions, and in the case of the G1FN and G2FN groups, after exposure to the smoke protocol. The sedentary animals were submitted to the same conditions as the swimming program, but in 10 cm of water for 15 minutes; this replicated the same stress resulting from exposure to water, but without the stimulus to swim.

During lactation (day 1, day 7, day 14, and day 21 of life) weight and nose-to-anus length were measured.

A digital electronic scale (Marte, model ASF11, Brazil) with a maximum capacity of 500g and minimum of 0.002g was used. Nose-to-anus length was measured using a millimeter ruler.

### Bone mineral density and bone mineral content

Forty-eight hours after the 30th training session, the offspring were euthanized with xylazine (40 mg/kg, IP) and ketamine (40 mg/kg, IP). After anesthesia was confirmed, approximately 1 ml of KCl 10% was injected into the left ventricle until cardiac arrest occurred in diastole. After death resulting from cardiac arrest in diastole was confirmed, the offspring underwent a surgical procedure to extract the femur and tibia of the right hind limb.

Bone mineral density and bone mineral content were measured using a densitometer (DPX-Alpha model, Lunar Corporation, Madison, USA) equipped with software for small animals.^4^

### Statistical analysis

The weight, length, BMD and BMC, and bone area of the femur and tibia were obtained from the offspring. A descriptive analysis was conducted, including means and standard deviation. Analysis of variance (one-way ANOVA) and Tukey's post-test were used to analyze BMC, BMD, and bone area. The results followed normal distribution (according to the Shapiro-Wilk test with 5% significance) and the groups were independent. All the analyses used Statistical Analysis System software (SAS).

## RESULTS

When the offspring were born, values for the parameter weight were not greater in the pups in the control group (G1) compared to the offspring of the breeding females exposed to tobacco smoke (G2) (p=0.890). This difference was not seen for the parameter body length (p=0.079).

Growth occurred in an unequal manner in the two groups of pups during lactation. During the entire lactation period, the body weights for pups in the G1 group were greater than the values for the offspring of the breeding females exposed to smoke (7, 14, and 21 days - p<0.001). No statistically significant difference was seen in body length at birth, with greater values for the offspring of the breeding females in group G1 compared to the offspring in group G2 (7 days - p=0.001, 14 and 21 days - p<0.001). (Table 1)

**Table 1:** Mean and standard deviation for weight and body length of offspring during lactation.

	G1	G2	P
Body weight			
Birth	6.67 ± 0.84	6.65 ± 0.67	0.890
7 days	12.50 ± 0.87	10.85 ± 1.69	<0.001
14 days	27.34 ± 4.60	21.87 ± 1.71	<0.001
21 days	34.13 ± 1.74	29.57 ± 2.55	<0.001
Body length			
Birth	5.20 ± 0.24	5.06 ± 0.35	0.079
7 days	6.46 ± 0.37	6.10 ± 0.45	<0.001
14 days	8.67 ± 0.79	7.76 ± 0.56	<0.001
21 days	10.43 ± 0.35	9.36 ± 0.71	<0.001

G1: breeding females, control group and G2: breeding females exposed to cigarette smoke.

The areas of the tibia and femur bones did not show a statistically significant difference according to ANOVA (p=0.058 and p=0.700, respectively). (Table 2)

**Table 2:** Mean and standard deviation of bone area.

		G1CS	G1CN	G1FS	G1FN
Area	Femur	1.34 ± 0.27	1.41 ± 0.17	1.31 ± 0.22	1.38 ± 0.22
	Tibia	1.1658 ± 0.21	1.15 ± 0.19	0.93 ± 0.16	1.13 ± 0.18
		G2CS	G2CN	G2FS	G2FN
Area	Femur	1.31 ± 0.14	1.43 ± 0.21	1.28 ± 0.09	1.53 ± 0.20
	Tibia	1.00 ± 0.10	0.91 ± 0.10	1.01 ± 0.13	1.00 ± 0.11

G1CS: offspring of breeding female control group, sedentary control; G1CN: offspring of breeding female control group, exercise control group; G1FS: offspring of breeding female control group, exposed to cigarette smoke and sedentary; G1FN: offspring of breeding female control group, exposed to cigarette smoke and exercise; G2CS: offspring of breeding females exposed to cigarette smoke, sedentary control group; G2CN: offspring of breeding females exposed to cigarette smoke, exercise control group; G2FS: offspring of breeding females exposed to cigarette smoke, exposed to cigarette smoke and sedentary; G2FN: offspring of breeding females exposed to cigarette smoke, exposed to cigarette smoke and exercise.

In the bone density study, no statistically significant difference was seen (p=0.366) between the study groups for the parameter bone mineral content for the femur. However, in the study this same parameter for the tibia showed lesser values for the G2CN group compared to the offspring in the G1CS (p=0.014), G1CN (p=0.023), and G1FN groups (p=0.008). (Table 3)

**Table 3:** Mean and standard deviation of bone mineral content.

		G1CS	G1CN	G1FS	G1FN
BMC	Femur	0.28 ± 0.05	0.29 ± 0.04	0.29 ± 0.05	0.29 ± 0.04
	Tibia	0.20 ± 0.03 ^a^	0.19 ± 0.03 ^b^	0.17 ± 0.02	0.19 ± 0.03 ^c^
		G2CS	G2CN	G2FS	G2FN
BMC	Femur	0.26 ± 0.03	0.28 ± 0.04	0.26 ± 0.02	0.30 ± 0.04
	Tibia	0.16 ± 0.02	0.15 ± 0.02	0.16 ± 0.02	0.16 ± 0.02

G1CS: offspring of breeding female control group, sedentary control; G1CN: offspring of breeding female control group, exercise control group; G1FS: offspring of breeding female control group, exposed to cigarette smoke and sedentary; G1FN: offspring of breeding female control group, exposed to cigarette smoke and exercise; G2CS: offspring of breeding females exposed to cigarette smoke, sedentary control group; G2CN: offspring of breeding females exposed to cigarette smoke, exercise control group; G2FS: offspring of breeding females exposed to cigarette smoke, exposed to cigarette smoke and sedentary; G2FN: offspring of breeding females exposed to cigarette smoke, exposed to cigarette smoke and exercise. Letters indicate statistical difference between: a = G1CS vs. G2CN (p=0.014), b = G1CN vs. G2CN (p=0.023), c = G1CN vs. G2CN (p=0.008).

When bone mineral density was analyzed, a difference was seen in the levels for the two bones which were studied. Measurement of bone mineral density in the femur showed greater density values for the G1FS group compared with the G2CS (p=0.004), G2CN (p<0.001), G2FS (p=0.008), and G2FN (p<0.001) groups. For the tibia, the animals in the G1FS group showed higher values compared to the G2CS (p=0.003) and G2FN (p=0.007) groups. (Table 4)

**Table 4:** Mean and standard deviation of bone mineral density.

		G1CS	G1CN	G1FS	G1FN
BMD	Femur	0.21 ± 0.01	0.20 ± 0.01	0.22 ± 0.02 ^a, b, c, d^	0.21 ± 0.01
	Tibia	0.17 ± 0.01	0.17 ± 0.02	0.18 ± 0.02 ^a, d^	0.17 ± 0.01
		G2CS	G2CN	G2FS	G2FN
BMD	Femur	0.20 ± 0.01	0.19 ± 0.01	0.20 ± 0.01	0.19 ± 0.01
	Tibia	0.16 ± 0.01	0.16 ± 0.01	0.16 ± 0.01	0.16 ± 0.01

G1CS: offspring of breeding female control group, sedentary control; G1CN: offspring of breeding female control group, exercise control group; G1FS: offspring of breeding female control group, exposed to cigarette smoke and sedentary; G1FN: offspring of breeding female control group, exposed to cigarette smoke and exercise; G2CS: offspring of breeding females exposed to cigarette smoke, sedentary control group; G2CN: offspring of breeding females exposed to cigarette smoke, exercise control group; G2FS: offspring of breeding females exposed to cigarette smoke, exposed to cigarette smoke and sedentary; G2FN: offspring of breeding females exposed to cigarette smoke, exposed to cigarette smoke and exercise. Letters indicate statistical difference between: a = G1FS vs. G2CS, b = G1FS vs. G2CN, b = G1FS vs. G2FS, b = G1FS vs. G2FN.

## DISCUSSION

After birth, the offspring of the breeding females exposed to passive cigarette smoke remained at lower weights, and over time the difference between this group and the offspring of the breeding females in the control group widened; this phenomenon may be explained by the low levels of prolactin which have been found in women who are active smokers during pregnancy and the post-natal period.^12^ ^,^ ^13^ Another study analyzed breastfeeding duration and found shorter periods of breastfeeding in children whose fathers were smokers.^13^ ^,^ ^14^

Similar to the findings of Gao et al.^15^ and Ino et al.^16^, in this study we also observed that the groups exposed to cigarette smoke continued to show lesser measurements in comparison with the control group. 

Lower BMD and BMC values were found in the offspring of breeding females exposed to cigarette smoke in comparisons with the offspring of the control rats. Gao et al.^4^ found similar results after 4 months of exposure to tobacco smoke. The literature shows that metabolic acidosis may negatively impact bone metabolism.^2^ In this study, the animals were exposed to passive smoking, which produces respiratory acidosis;^6^ this may suggest that the changes seen in BMD and BMC may be connected to the effects of smoke exposure.

It has already been shown that gestation and the neonatal period have a greater influence on individual growth and health,^16^ and this present study confirmed that maternal exposure to passive cigarette smoke was a determining factor in reduced values for bone mineral density and content. 

According to Gao et al.,^4^ smoking suppresses the bone formation and increases bone reabsorption. 

Exposure during growth led to significant differences in animal weight and length. The study by Gao et al.^15^ found a statistically significant difference only after 4 months of exposure; the rats which were exposed for 2 or 3 months did not exhibit differences in the bone parameters studied.^4^

With regard to the exercise variable, no alterations were seen that could be attributed to exercise; this lack of response is likely due to the protocol period (30 sessions). According to Iwamoto et al.,^17^ osteopenic rats did not show a statistically significant difference in the parameter BMD in 4 or 8 weeks of exercise; a difference was only seen after 12 weeks.

Another fact that can be considered is that bone maturation only occurs after the 8th month of life in rats,^4^ indicating a future possibility to investigate growth after the 8th month to assess the extent of the effect passive chronic smoking associated with swimming exercise has on a larger group of animals.

## CONCLUSION

In summary, in this study the model of male rats exposed to passive smoking during fetal development exhibited a marked decrease in weight and length. Swimming exercise did not exhibit a significant impact on the parameters analyzed in this study.

In the bone tissue, maternal exposure to passive smoking was a determining factor for changes in the BMD and BMC of the offspring.
